# YIM-P59. Relationships between the Th17 and innate lymphoid cell signature in enthesitis related arthritis

**DOI:** 10.1186/1546-0096-12-S1-Y6

**Published:** 2014-09-17

**Authors:** Hannah Lom, Kiran Nistala, David Bending, Yiannis Ioannou, Mona Bajaj-Elliott, Lucy Wedderburn

**Affiliations:** 1 Infection, Inflammation and Rheumatology Section, UCL Institute of Child Health, London, UK; 2 Arthritis Research UK Centre for Adolescent Rheumatology, London, UK; 3 Centre for Rheumatology, University College London, London, UK; 4 Adolescent and Adult Rheumatology, Great Ormond St Hospital & University College London Hospitals NHS Foundation Trusts, London, UK

## Introduction

Enthesitis related arthritis (ERA), a subtype of juvenile idiopathic arthritis (JIA) has a close association with HLA-B27. Previous studies suggest a role for Th17 cells in disease pathogenesis; at present the link between Th17 immunity and HLA-B27 is unknown. Innate lymphoid cells (ILCs) form a bridge between the innate and adaptive immune systems, producing signature cytokines in response to cytokine stimulation. ILCs lack common surface markers associated with known leukocyte lineages, but constitutively express the IL-7 receptor-α(CD127). ILCs can be divided into 3 types (1, 2 and 3) which mimic the T cell subsets (Th1, Th2 and Th17) based on their transcription factor and cytokine profiles.

## Objectives

To investigate Th17 and ILC populations in the joint and blood of ERA JIA patients.

## Methods

Th17 cells and ILCs from the peripheral blood mononuclear cells (PBMC) of healthy controls and ERA patients and synovial fluid mononuclear cells (SFMC) of ERA patients were analysed by flow cytometry (n=7). Th17 cells were identified as CD3 positive CD4 positive T cells which secreted interleukin (IL)-17 after stimulation with PMA and ionomycin . ILCs were identified as lineage (CD3, αβTCR, γδTCR, CD19, CD14, CD11c, CD16, CD1a, CD34, CD94, BDCA2 and FcεRIα) negative, CD45 positive, CD127 positive cells.

## Results

Th17 cells (CD3+CD4+IL-17+) were enriched in the joint compared to the blood of ERA patients and healthy blood (p=0.0446). Within the Th17 populations, an increase of IL-17/IFNγ double-producing cells was found in the joint compared to the blood of ERA patients. Compared to available data in other types of JIA, the number of Th17 cells was higher in ERA (median = 4.1%) than other JIA subtypes, including extended oligoarthritis (median = 1.99%). An enrichment of ILCs was observed in the joint of ERA patients (0.15-0.5% of lymphocytes) compared to healthy blood (<0.1%) (p=0.0114). Interestingly, a positive correlation between Th17 and ILC numbers in the joint of ERA patients was demonstrated (r=0.6).

## Conclusion

These data show that Th17 cells and ILCs are enriched in the joints of ERA JIA patients. Further work is warranted to explore the interplay between these two immune cell populations in the context of ERA.

## Disclosure of interest

None declared

